# Direct RNA-Based Detection and Differentiation of CTX-M-Type Extended-Spectrum β-Lactamases (ESBL)

**DOI:** 10.1371/journal.pone.0080079

**Published:** 2013-11-05

**Authors:** Claudia Stein, Oliwia Makarewicz, Yvonne Pfeifer, Christian Brandt, João Costa Ramos, Mareike Klinger, Mathias W. Pletz

**Affiliations:** 1 Center for Infectious Diseases and Infection’s Control, Jena University Hospital, Jena, Germany; 2 Center for Sepsis Control and Care, Jena University Hospital, Jena, Germany; 3 Nosocomial pathogens and antibiotic resistance, Robert Koch Institute, Wernigerode, Germany; University of Malaya, Malaysia

## Abstract

The current global spread of multi-resistant Gram-negatives, particularly extended spectrum β-lactamases expressing bacteria, increases the likelihood of inappropriate empiric treatment of critically ill patients with subsequently increased mortality. From a clinical perspective, fast detection of resistant pathogens would allow a pre-emptive correction of an initially inappropriate treatment. Here we present diagnostic amplification-sequencing approach as proof of principal based on the fast molecular detection and correct discrimination of CTX-M-β-lactamases, the most frequent ESBL family. The workflow consists of the isolation of total mRNA and CTX-M-specific reverse transcription (RT), amplification and pyrosequencing. Due to the high variability of the CTX-M-β-lactamase-genes, degenerated primers for RT, qRT as well as for pyrosequencing, were used and the suitability and discriminatory performance of two conserved positions within the CTX-M genes were analyzed, using one protocol for all isolates and positions, respectively. Using this approach, no information regarding the expected CTX-M variant is needed since all sequences are covered by these degenerated primers. The presented workflow can be conducted within eight hours and has the potential to be expanded to other β-lactamase families.

## Introduction

The number of ESBLs of the CTX-M-type has increased dramatically in the past ten years, and the majority of 3^rd^ generation cephalosporin resistant clinical isolates of *Escherichia coli* and *Klebsiella pneumoniae* produce these enzymes [1]. Besides CTX-M, clinical isolates often harbor many other β-lactamases (e.g. TEM, SHV, OXA). The increase of CTX-M-producers has led to an increasing use of carbapenems resulting in the emergence of carbapenem resistant *Enterobacteriaceae*.

The CTX-M enzymes belong to class A of the serine-β-lactamases and were named in reference to their uncommon preference to hydrolyze cefotaxime and cefepime more effectively than ceftazidime [2]. However, in the past years, CTX-M ESBLs, with highly increased hydrolyzing activity against ceftazidime (e.g. CTX-M-15), have been frequently observed in human and animal isolates as well as in environmental samples [3,4,5]. Based on the phylogenetic properties approximately 120 CTX-M variants are described so far, that cluster into five main groups [6].

Nucleic acid testing (NAT) based detection of CTX-M β-lactamases is fast but challenging, because to our knowledge non of the commercially available NAT-based tests for bacterial infections is able to detect all CTX-M variants [7]. They focus instead on species determinants and the detection of some individual frequent CTX-M variants. Such an approach is of limited use because CTX-M evolution, i.e. the rise of novel variants and composition of regional circulating CTX-M variants, are subjects to a dynamic change and the epidemiology exhibits large regional differences [8].

The spread of Gram-negative bacteria producing extended-spectrum β-lactamases (ESBL) has become a rising problem worldwide. Several clinical studies have shown that blood stream infections with enterobacteria expressing ESBL are associated with an increase in mortality rates [9,10]. This increased mortality is not caused by an enhanced virulence of the culprit pathogen, but by initial inappropriate treatment resulting in clinical failure [11,12]. Empiric treatment suggested by most sepsis guidelines (i.e. β-lactam with antipseudomonal activity, in optional combination with an aminoglycoside or a fluoroquinolone) does not always cover ESBL producers [13,14,15,16]. 

Since blood culture results are available only after 2 - 4 days and a delay in antibiotic treatment by more than 1 hour is already associated with an increase in mortality in critically ill patients [17], guidelines recommend that empiric treatment should be based on local resistance rates and individual patient risk factors [18]. However, these “risk factors” (i.e. prior antimicrobial treatment, hospitalization for > 4 days, etc.) are poorly defined, and may result in inappropriate treatment for the individual patient. Therefore an early detection of ESBL expressing pathogens is crucial for individual patient outcome. 

PCR-based determination of β-lactamases is facing the following challenges: i) some types exhibit strong sequence discrepancies, disabling primer-annealing within one type; ii) different CTX-M variants exhibit different substrate spectra, therefore discrimination to the type level is required; iii) the presence of more than one CTX-M variant within the same bacterium and iv) detection of the gene does not necessarily mean expression of the enzyme [19,20]. 

These obstacles could be resolved by a strategy combining reverse transcription of mRNA and subsequent amplification, followed by fast sequencing of a specific mutation hot spot that will allow discrimination of CTX-M-types on single nucleotide level. However, some CTX-M-variants exhibit substitutions that spread nearly over the entire sequence. Therefore, a comprehensive identification of all relevant substitutions and sequence homologies related to phenotypic resistance is needed to allocate the appropriate regions for PCR and pyrosequencing, and to develop PCR conditions that are feasible for a transfer into clinical routine testing. Here we present our efforts to identify adequate regions within the huge and steadily increasing number of CTX-M variants that allow a fast detection and accurate identification of individual CTX-M variants.

## Materials and Methods

### Bacterial strains and antimicrobial susceptibility testing

Bacterial strains used in this work were mainly clinical isolates (n=16) of *Enterobacteriaceae*, with resistance to 3^rd^ generation cephalosporin’s and/or carbapenems (Table 1). These strains were pre-characterised in the Robert-Koch-Institute using PCR and sequencing of various β-lactamase genes (*bla*_TEM-type_, *bla*_SHV-type_, *bla*_CTX-M-type_, *bla*_OXA-type_, *bla*_NDM-type_) as described previously [21,22,23]. The three control strains contained other β-lactamases than CTX-M, including one *E. coli* strain CS01 bearing the TEM-2 β-lactamase, and that was constructed by transformation of the pET15b plasmid into Ca^2+^-competent JM109 cells, as described previously [24]. We also analyzed the specificity of the method, by detection and identification of CTX-M transcripts derived from strains bearing up to 7 different β-lactamases, which encompass most common variants.

**Table 1:** Bacterial strains used in this study.

**Isolate's name**	**Species**	**Target CTX-M**	**Other β-lactamases**
RKI 26/08	*E. coli*	CTX-M-1	
RKI 443/08	*E. coli*	CTX-M-14	
RKI 25/08	*E. coli*	CTX-M-15	
RKI 427/08	*E. coli*	CTX-M-27	TEM-1
RKI 29/04	*E. coli*	CTX-M-3	
RKI 128/04	*E. coli*	CTX-M-2	
CS 01	*E. coli*		TEM-2
RKI 2/10*	*E. coli*	CTX-M-15	TEM-1, OXA-1, OXA-2, NDM-1
RKI 209/10*	*S. enterica*	CTX-M-8	
RKI *K*.p. 36 SHV	*K. pneumoniae*		SHV-4
RKI 28/08	*K. pneumoniae*		OXA-10, SHV-5
RKI 93/07	*K. pneumoniae*	CTX-M-15	TEM-1, SHV-28, CTX-M-15, OXA-1, OXA-9, CMY-like, NDM-1
RKI 346/12	*K. pneumoniae*	CTX-M-15	TEM-1, SHV-1, OXA-1, OXA-9, OXA-48
RKI VW823	*E. cloacae*	CTX-M-25	SHV-11, TEM-1
RKI 428/08	*E. cloacae*	CTX-M-9	
RKI 1/10*	*E. cloacae*	CTX-M-15	TEM-1, OXA-1, OXA-48
RKI 181/13	*K. oxytoca*		OXY

* Further characteristics of these isolates were described in other studies [21,23,37,38]

Determination of antimicrobial susceptibilities to carbapenems (ertapenem, imipenem, meropenem), and 12 further antibiotics (ampicillin, cefotaxime, ceftazidime, cefoxitin, nalidixic acid, ciprofloxacin, gentamicin, amikacin, streptomycin, chloramphenicol, tetracycline and trimethoprim/sulfamethoxazole), were performed by Etest (bioMérieux, Nuertingen, Germany) and microbroth dilution, respectively, according to the CLSI criteria [25]. 

### RNA isolation

All strains were grown in Mueller-Hinton (MH) medium (Roth) supplied with 16 μg/ml cefotaxime (Fresenius Kabi) or 100 μg/ml ampicillin (Roth) at 37 °C for 5 hours. A culture volume of 3 ml was directly used for total RNA isolation.

Bacterial RNA was extracted using a Tempus^TM^ Blood RNA Tube and Tempus^TM^ Spin RNA Isolation Kit according to the manufacturer instructions (Life Technologies). DNase digestion was carried out using AbsoluteRNA Wash Solution (Life Technologies) on the column. After elution with RNase-free water (Fermentas) RNA samples were stored at -80 °C. RNA quality and quantity were analyzed using an Agilent Bioanalyzer 2100 (Agilent Technologies). All reagents were obtained from Agilent Technologies and measurements were performed according to manufacture’s protocol. The RNA samples were diluted in RNase-free water to a final concentration of about 2 ng/µl, and denaturized at 72 °C for 3 min. Pico RNA Chip and the Prokaryote Total RNA Pico assay were used to quantify total RNA and to determine the RIN value, expressing the quality of the prepared RNA based on 16S and 23S rRNA. For the further analysis only RNA samples were used that exhibited a RIN > 7.5.

### Reverse Transcription (RT)

Corresponding to specific resistance pattern we used degenerated primers for reverse transcription, PCR and pyrosequencing. The annealing-reactions were performed in 20 µl volume containing 10 ng of total RNA and 20 µM of degenerated and 5'-biotinylated reverse primers *rev*_CTX-M314_ (TCVGCAATSGGATTRTAGTTAAYMA) and *rev*_CTX-M739_ (GCCARATVACCGCRATATCRTT) corresponding to positions +314 to +339 and +739 to +761, respectively. Each reaction mix was incubated for 6 min at 72 °C and cooled down to 37 °C in a thermo-cycler. After addition of the RT-master-mix, the samples were incubated at 37 °C for 60 min, followed by the denaturation step at 94 °C for 10 min. RT-master-mix was composed of 0.167 mM dNTPs each (Roth), 0.6 U/µl RNase Inhibitor (Life Technologies), 1 x reaction buffer supplied by manufacturer (Sensiscript RT Kit, Qiagen), 0.33 U/µl Sensiscript (Sensiscript RT Kit, Qiagen), and 0.1 mg/ml BSA (Life Technologies).

### Real-Time PCR

**Figure 1:**
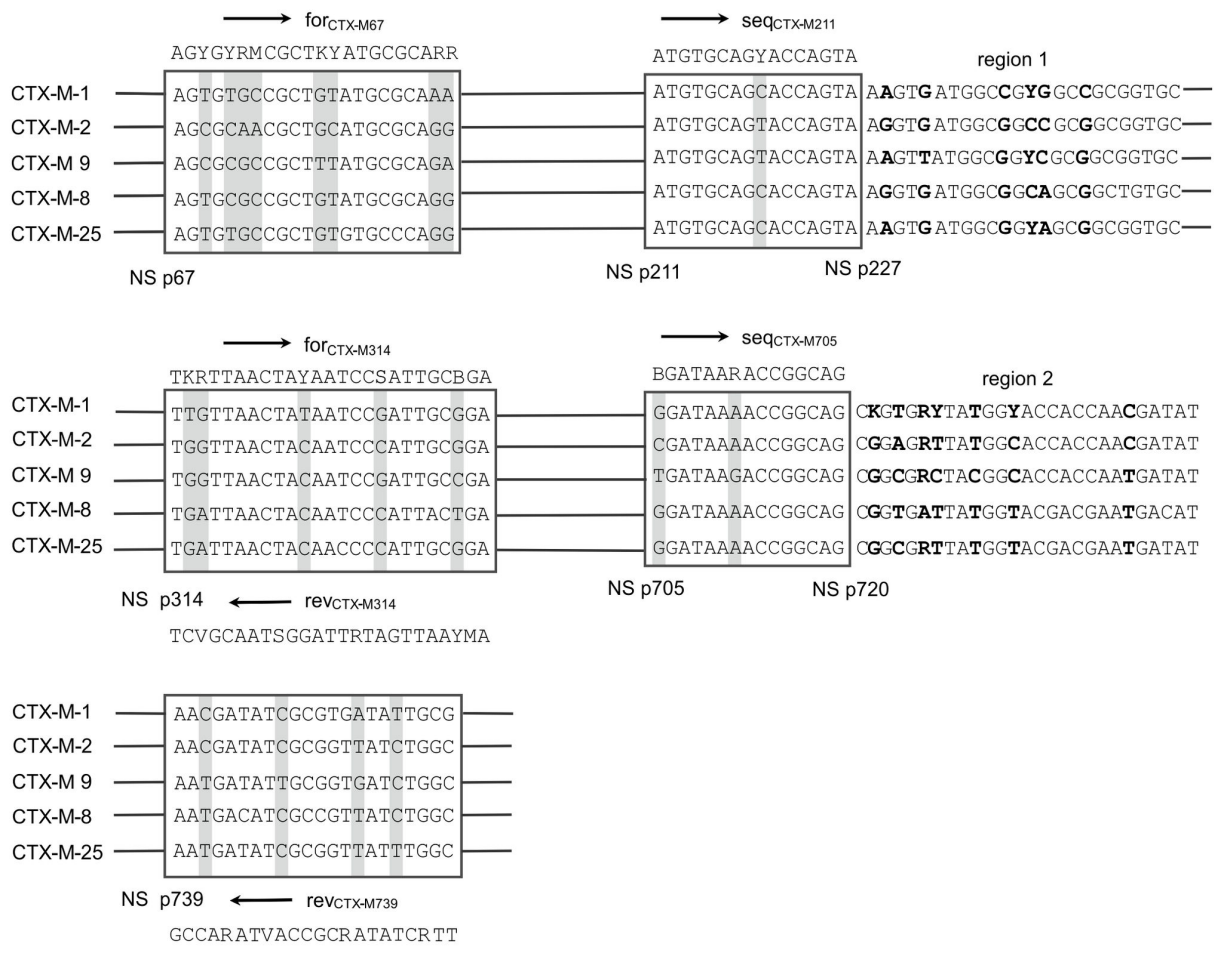
Alignment of representatives of the CTX-M groups. The IUPAC code was used to highlight the variable nucleotides within the primer sequences and the sequence positions analyzed by pyrosequencing, for the last the code displays the nucleotide variants within the group (for example K in group 1 = G or T). Positions of PCR and sequencing primers (for details see Material and Methods) are framed and the sense and antisense directions are indicated with arrows. The degenerated nucleotides within the primer annealing positions are highlighted in grey.

Prior to the qRT, we used 10 ng total RNA prepared from clinical ESBL isolates (Table 1) bearing members of the five phylogenetic CTX-M groups, as well as 13 related serine-β-lactamases of different classes (TEM-1, TEM-2, SHV-1, SHV-4, SHV-5, SHV-28, OXA-1, OXA-2, OXA-9, OXA-10, OXA-48, CMY, AmpC) and one metallo-β-lactamase (NDM-1), to produce two cDNA transcripts by reverse transcription using degenerated primers *rev*_CTX-M314_ and *rev*_CTX-M739_. The cDNA transcripts were amplified by qRT using degenerated PCR-primer sets *for*_CTX-M67_ / *rev*_CTX-M314_ and *for*_CTX-M314_ / *rev*_CTX-M739_. The primers were designed based on conserved sequence regions of all CTX-M variants (for details see Figure 1). The quantitative real-time PCR (qRT) was performed in a Rotor-GeneQ cycler (Qiagen), by directly applying 2 µl of the RT reaction and the degenerated primer sets *bla*_CTX-M67_ (AGYGYRMCGCTKYATGCGCARR, corresponding to position +67 to +89) and *rev*_CTX-M314_ as well as *for*_CTX-M314_ (TKRTTAACTAYAATCCSATTGCBGA) corresponding to position +314 to +339 and *rev*_CTX-M739_. Both reverse primers were initially used for reverse transcription. PCR reaction mixture was composed of 1.8 mM MgCl_2_, 1 x PCR buffer (Life technologies), 0.2 mM dNTPs (Roth), 0.6 µM of each primer (Sigma Aldrich), 0.15x SybrGreen (Life Technologies), 0.08 U/µl Platinum Taq DNA Polymerase (Life Technologies), and 0.1 mg/ml BSA (Life Technologies). The PCR was run as follows: pre-denaturation at 99 °C for 10 s and 95 °C for 50 s; 45 cycles composed of 95 °C for 20 s, 51 °C for 20 s and 72 °C for 20 s. The melting temperatures of PCR-products were determined by stepwise increasing (0.5 °C / 4 s) of the temperature (from 75 °C to 99 °C). A linearized plasmidal CTX-M-15 gene was used as a molecular copy standard.

### Pyrosequencing

Pyrosequencing analysis was performed on the PyroMark Q24 (Qiagen). A PCR reaction volume of 20 µl was added to the pyrosequencing reaction mixture (PyroMark Gold Q24 reagent, Qiagen). The CTX-M sequence was analyzed using the sequencing primer *seq*_CTX-M211_ (ATGTGCAGYACCAGTA) or *seq*_CTX-M705_ (BGATAARACCGGCAG) annealing at positions +211 to +226, or +705 to +720, respectively. All remaining reagents were provided by the manufacturer (PyroMark Gold Q24 reagents, Qiagen), and measurement was performed according the manufactures protocol. 

### Sequence alignment

In total, 120 *bla*_CTX-M_ gene sequences available online at the National Center for Biotechnology Information (NCBI) could be analyzed using DS Gene 1.5 software (Accelrys Ltd). The nucleic acid sequences were aligned according to the multiple alignment method ClustalW (open gap penalty 10, extended gap penalty 5). For the phylogenetic calculations we used the tree building method TreeDyn [26] (construction: PhyML).

## Results and Discussion

### Lineage of CTX-M types and unambiguous mutations’ positions

Referring to nucleic acid homologies, gene sequences of all known CTX-M-variants (n=120) were extracted from the gene bank (NCBI), aligned and displayed in a phylogenetic tree, that revealed the expected relationships by dividing the CTX-M-variants into the known 5 lineages [27] (for details see Figure S1). In total, CTX-M group 9 contains 40 closely related sequences, within which CTX-M-9, -14 and CTX-M-27 are the main clinically relevant variants [28]. CTX-M group 1 involves 46 enzyme variants, including the widespread and clinically relevant CTX-M-1, -3, -15 and CTX-M-32 [29]. CTX-M group 2 includes 19 sequences with CTX-M-2 representing the most common β-lactamase [6]. Group 8 and group 25 contain only 3 and 9 enzyme variants of minor clinical importance. Detailed reviews on clinically relevant CTX-Ms were published elsewhere [29,30,31]. Two variants, CTX-M-74 and -75, are not assigned to any group, but they seem to be closely related to CTX-M group 2.

Based on the alignments, we found one sequence position (+227 to +246, relative to ATG) appropriate for discrimination between all phylogenetic groups of the CTX-M β-lactamases. A second region (+720 to +747), corresponding to amino acid position 240 that plays an important role in the resistance pattern of CTX-Ms [32], was chosen for further discrimination of the variants within the groups. 

### Amplification and specificity of qRT products

45 cycles were run to ensure that all reactions reached saturation, and sufficient PCR product concentrations were obtained for the following pyrosequencing reaction (see Figures S2 and S3). Melting temperatures (Tm) of the CTX-M products were determined, indicating similar values within the groups (for details Table 2 and 3). Since similar melting temperatures were also obtained for β-lactamases of the SHV-, TEM- and OXA-families that were used as unspecific controls, a melting curve analysis was unable to distinguish between CTX-M and non CTX-M-types. Compared to the SHV-, TEM-, OXY- and OXA-types, the c_t_ values (cycle number at threshold) for CTX-M-products were lower and the PCR-products of non-CTX-M variants crossed the threshold later, indicating unspecific PCR-products. However, since c_t_ values strongly depend on template concentration, they are not sufficient for discrimination between specific and non-specific targets. 

**Table 2:** Analysis of region 1.

	**RNA isolation**		**qRT parameters**		**NP 1**		**NP 2**		**NP 3**			**NP 4**		**NP 5**		**NP 6**	
**Enzyme**	C_RNA_	**RIN**	**C_t_**	**T_m_**	**A**	**G**	**G**	**T**	**(A)**	**C**	**G**	**C**	**T**	**A**	**G**	**C**	**G**
CTX-M-1	23.7	8.9	19.57	89.25	100	0	100	0	10	90*	0	3	97*	8	92	100*	0
CTX-M-3	20.1	8	20.93	89.75	100	0	99	1	11	89*	0	68	32	9	91	96*	4
CTX-M-15	230	n.d.	17.4	89.9	100	0	99	1	13	97*	0	73	27	13	87	96*	4
CTX-M-2	61.2	8.1	22.44	91,00	24	76*	94	6	12	19	69	78	22	11	89	28	72
CTX-M-9	34.8	8.3	25.7	91.6	100	0	37	63*	10	30	60	63	37	20	80	36	64
CTX-M-14	48.4	9.3	22.54	91.5	100	0	59	41*	4	58	38	57	43	4	96	56	44
CTX-M-27	49.3	9	21.87	91.4	100	0	60	40*	3	60	36	52	48	4	96	63	37
CTX-M-8	26,0	8	25.83	91.6	0	100*	100	0	2	0	98*	89	11	89	11	0	100*
CTX-M-25	43.9	8	29.02	90.25	100	0	100	0	4	0	96*	90	10	94	6	1	99*
SHV-4	80	n.d.	35.41	92.85	n.d.	n.d.	n.d.	n.d.	n.d.	n.d.	n.d.	n.d.	n.d.	n.d.	n.d.	n.d.	n.d.
TEM-2	45.1	n.d.	31.35	89.15	n.d.	n.d.	n.d.	n.d.	n.d.	n.d.	n.d.	n.d.	n.d.	n.d.	n.d.	n.d.	n.d.
OXA-10	44.1	n.d.	32.01	92.6	n.d.	n.d.	n.d.	n.d.	n.d.	n.d.	n.d.	n.d.	n.d.	n.d.	n.d.	n.d.	n.d.

n.d. = not detectable

* Nucleotide evidences discriminative for the corresponding nucleotide position

Parameters (concentration and RIN quality control) of the total RNA isolation, real-time PCR (c_t_ value and melting temperature T_m_) and substitutional analysis of the nucleotide positions (NP) 1 to 6 of the pyrograms given as percent (%) of evidence.

**Table 3:** Analysis of region 2.

	**qRT parameters**		**NP 1**		**NP 2**			**NP 3**		**NP 4**		**NP 5**		**NP 6**		**NP 7**	
**Enzyme**	**C_t_**	**T_m_**	**G**	**T**	**A**	**C**	**T**	**A**	**G**	**C**	**T**	**C**	**T**	**C**	**T**	**C**	**T**
CTX-M-1	16.82	94.0	87	13	6	11	83*	91*	9	96	4	14	86*	87	13	67	33
CTX-M-3	18.12	94.3	86	14	7	13	80*	92*	8	94	6	17	83*	85	15	65	35
CTX-M-15	17.14	94.5	96	4	3	8	89*	5	95*	98	2	10	90*	89	11	72	28
CTX-M-2	14.16	94.0	88	12	73*	14	13	94	6	16	84	10	90	83	17	64	36
CTX-M-9	22.71	95.8	90	10	6	59	35	95*	5	100*	0	62	38	90	10	68	32
CTX-M-14	19.69	96.0	89	11	5	46	49	94*	6	99*	1	49	51	91	3	68	32
CTX-M-27	19.12	96.0	93	7	4	45	51	55	45*	100*	0	46	54	90	10	72	28
CTX-M-8	30.14	92.5	53	47	18	36	46	91	9	60	40	45	55	39	61	47	53
CTX-M-25	22.44	93.5	96	4	0	86*	14	0	100	100	0	92	8	86	14	80	20
SHV-4	21.44	91.5	n.d.	n.d.	n.d.	n.d.	n.d.	n.d.	n.d.	n.d.	n.d.	n.d.	n.d.	n.d.	n.d.	n.d.	n.d.
TEM-2	30.78	87.6 (84.9)^#^	n.d.	n.d.	n.d.	n.d.	n.d.	n.d.	n.d.	n.d.	n.d.	n.d.	n.d.	n.d.	n.d.	n.d.	n.d.
OXA-10	25.89	92.7 (89.7)^#^	n.d.	n.d.	n.d.	n.d.	n.d.	n.d.	n.d.	n.d.	n.d.	n.d.	n.d.	n.d.	n.d.	n.d.	n.d.

* Nucleotide evidences discriminative for the corresponding nucleotide position

^#^ Melting peak of a second PCR-product

Parameters of the real-time PCR (c_t_ value and melting temperature T_m_), and substitutional analysis of the nucleotide positions (NP) 1 to 7 of the pyrograms given as percent (%) of evidence.

**Figure 2:**
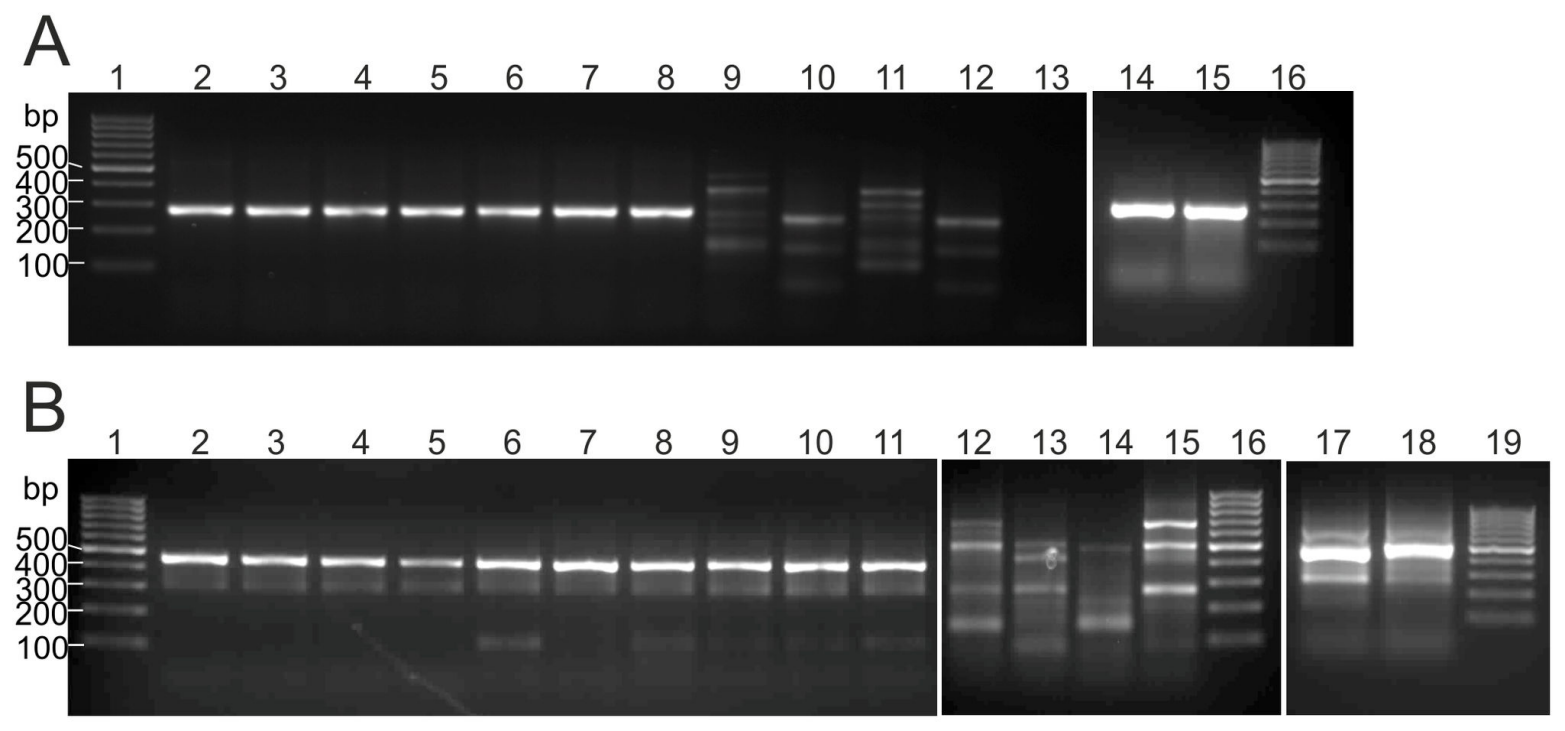
PCR products of various β-lactamases. The qRT samples were analyzed on a 1.5 % agarose gel after 45 cycles PCR. (A) PCR-products of NP- region 1; Lines: 1, 16= Low-range molecular ladder (Thermo Scientific), 2 = CTX-M-15, 3 = CTX-M-1, 4 = CTX-M-2, 5 = CTX-M-3, 6 = CTX-M-9, 7 = CTX-M-14, 8 = CTX-M-27, 9 = SHV-4, 10 = TEM-1, 11 = OXA-10, 12 = TEM-1, 13 = NTC, 14 = CTX-M-8, 15 = CTX-M-25. (B) PCR-products of NP-region 2; 1, 16 and 19 = Low-range molecular ladder (Thermo Scientific), 2 = 1 pg CTX-M-15, 3 = 10^-1^ pg CTX-M-15, 4 = 10^-2^ pg CTX-M-15, 5 = 10^-3^ pg CTX-M-15, 6 = CTX-M-1, 7 = CTX-M-2, 8 = CTX-M-3, 9 = CTX-M-9, 10 = CTX-M-14, 11 = CTX-M-27, 12 = OXA-1, 13 = OXA-10, 14 = TEM-2, 15 = SHV-4, 17 = CTX-M-8, 18 = CTX-M-25.

Therefore, the specificity of the PCR-products was further analyzed using agarose gel electrophoresis. Using the degenerated primer sets mentioned above, only the CTX-M-variants were successfully transcribed to cDNA and amplified to specific products of 268 bp and 447 bp (Figure 2). The band patterns of obtained amplicons for TEM-3, SHV-5, and OXA-10 contained many weak signals, indicating that these degenerated primers annealed only non-specifically under the given RT and qRT conditions. 

### Quality of the pyrograms

For sufficient interpretability, the required signal peaks have to be twenty fold higher than the background signals. In general, signal-to-noise ratio of the pyrograms obtained for the region 1 (nucleic acid region +227 to +246) were of higher quality, and the individual peaks could be clearly discriminated for all CTX-M-types, independently of the phylogenetic group. Pyrograms of region 2 (nucleic acid region +720 to +746) were of minor quality compared to region 1, but were still evaluable. The program signals of all nucleotide positions (NPs) allowed satisfying discrimination between groups 1, 2, and 9. Groups 8 and 25 showed well evaluable peaks for NP 1 - NP 3. The non-CTX-M β-lactamases (SHV, TEM, OXA) yielded very weak and non-evaluable peaks.

The pyrograms of region 1 of the mixed samples containing various β-lactamases all exhibited good peak quality and quantity and could be clearly discriminated. 

### CTX-M group differentiation based on region 1

**Figure 3:**
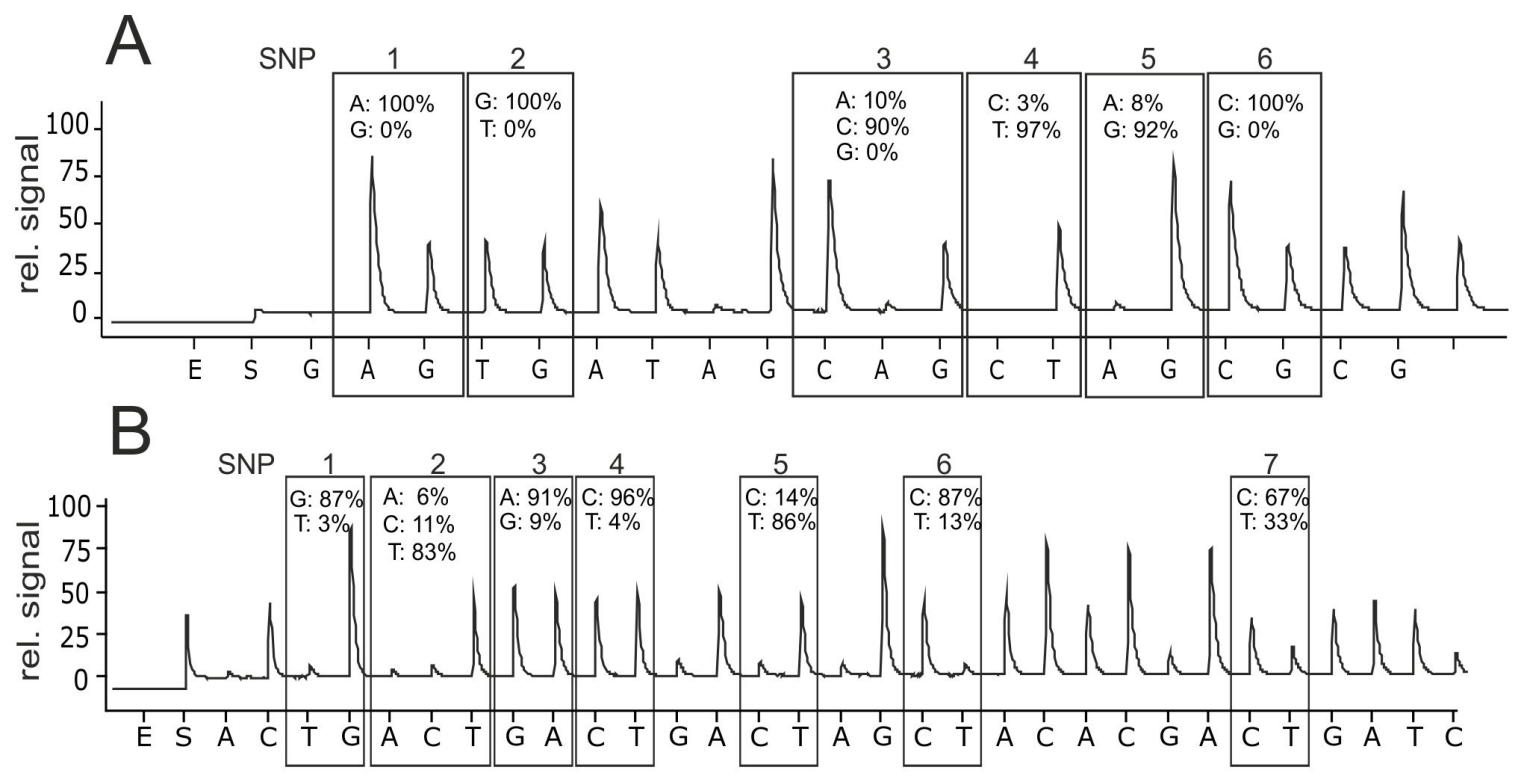
Representative pyrograms of CTX-M-1. Sequence obtained by degenerated primer *seq*_CTX-M211_ (A) and *seq*_CTX-M705_ (B). The sequentially applied nucleotides are given at the X-axis, alternative NPs are boxed and the bases are indicated on the top. The Y-axis shows the relative signal intensity. The probabilities of the nucleotides were calculated by the PyroMark-evaluation software in percent.

In general, protocols for pyrosequencing are usually designed for determination of only one or few substitutions within a short sequence. However, in many CTX-M variants, substitutions are distributed over the entire gene. For the chosen discriminating region, ranging from +227 to +246 (Figure 1), we developed an optimized sequential application of nucleotides including an unspecific adenine at nucleotide position (NP) 4 that allowed a clear resolution of this position bearing a high GC-content (for details see Figure 3). The sequencing primer *seq*_CTX-M211_ was also designed as a degenerated oligonucleotide. The read outs were evaluated by the PyroMark Assay Design 2.0. Ink software. The probabilities of the incorporated nucleotides at the positions of interest are displayed as percentages (see Table 2). Specific distribution patterns could be allocated to the individual groups: Cytosine at NP 3 and NP 6 with an evidence of >90 % was unique for group 1 members. Thymine at NP 4 differentiates the clinically relevant CTX-M-1 and -32 and further variants (114, 116, 23, 52, 53, 55, 57, 60, 61, 69, and 79) from the other representatives of group 1. Groups 8 and 25 showed unique distributions for guanine at NP 3 and NP 6, simultaneously differing at NP 1 with guanine for group 8 (100 %) and adenine for group 25 (100 %). Variants of group 9 could be identified by the thymine at NP 2 since group 1, 2, 8, and 25 exhibited strong preferences to guanine (>94 %). Group 2 could be distinguished from other groups by the presence of guanine at NP 1 and NP 5, even when the evidence was only 76 % or 89 %, respectively. Guanine at NP 5 separated group 2 from group 8 (adenine).

The non-CTX-M β-lactamases (SHV, TEM, OXA and NDM) were not detected by the sequencing reaction even though unspecific PCR products were generated in the qRT. 

### Differentiation of CTX-M variants based on region 2

By sequencing a second short region ranking from +720 to +746, a more detailed discrimination of the variants of group 1, 9, and 25 could be obtained (Table 3). This position was already described as discriminative for some subgroups by Naas et al. [33]. Guanine at NP 3 (+726) corresponds to Asp240Gly that confers increased ceftazidime resistance [34]. 

The sequences of group 1 showed the highest variance within region 2, CTX-M-34 has a guanine at NP 1 and a thymine at NP 4. Thymine at NP 4 was also present in CTX-M-10, -37 and -53. Guanine at NP 3 was present within group 1 in many variants, of which some are highly clinically relevant. Within group 9, guanines were found in CTX-M-16, 93, 121, 105, 98, 102, and -27, within group 2 in CTX-M-131 and -43, and within group 25 in CTX-M-25, 94, 100, and -41. In summary, presence of guanine at this position allowed discrimination of the ceftazidime resistant CTX-M-phenotype. 

Adenine at NP 2 (73 %) and thymine at NP 4 (84 %) allowed a clear assignment to group 2. Thymine at NP 2 was unique for group 1, whereas cytosine at NP 2 allocated variants to group 25 and group 9. In summary, region 2 yielded additional information according the group allocation for 1, 2, 9 and 25.

### Discriminative accuracy by coexistence of CTX-M with other β-lactamases

**Figure 4:**
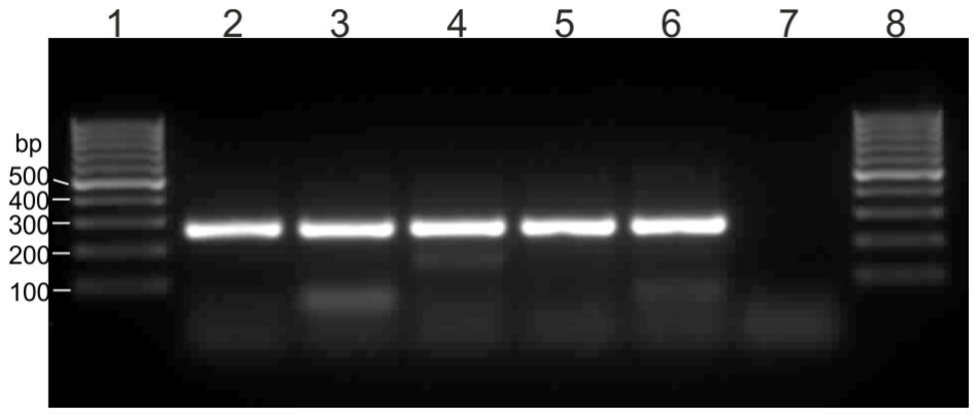
PCR-products of mixed β-lactamase samples. The qRT samples were analyzed on a 1.5 % agarose gel after 45 cycles PCR. (A) PCR-products of region 1; Lines: 1, 8 = Low-range molecular ladder (Thermo Scientific), 2 = CTX-M-15, 3 = RKI 01/10, 4 = RKI 02/10, 5 = RKI 93/07, 6 = RKI 364/12 .

To test the performance of the amplification-sequencing method under “real life” conditions, we analyzed the efficiency to discriminate the CTX-M-15 gene within various clinical isolates, which also contained up to 6 other β-lactamase genes of non-CTX-M type. Our results clearly showed that the distribution of the incorporated nucleotides at all NPs was similar to that of the control strain solely harboring CTX-M-15, indicating that the presence of non-CTX-M lactamases does not disturb the efficiency of the degenerated primers (Table 4). Moreover, the primer sets produced only specific amplicons when CTX-M genes were present simultaneously with non-CTX-M genes (Figure 4). (Contrary, when no CTX-M gene was present some unspecific PCR products could be determined (Figure 2)). Due to the common co-existence of different β-lactamases in clinical isolates, this robustness is crucial for clinical applicability.

**Table 4:** Substitutional analysis of region 1 in the presence of non-CTX-M β-lactamases.

	**qRT parameters**		**NP 1**		**NP 2**		**NP 3**			**NP 4**		**NP 5**		**NP 6**	
**Enzyme**	**C_t_**	**T_m_**	**A**	**G**	**G**	**T**	**A**	**C**	**G**	**C**	**T**	**A**	**G**	**C**	**G**
CTX-M-15	24.63	89.75	98	2	100	0	3	97	0	89	11	4	96	88	12
RKI 01/10	18.90	89.65	98	2	100	0	3	97	0	89	11	4	96	91	9
RKI 02/10	17.82	89.65	99	1	100	0	2	98	0	90	10	4	96	95	5
RKI 93/07	20.50	89.65	99	1	100	0	2	98	0	90	10	3	97	89	11
RKI 364/12	17.74	89.65	97	3	100	0	5	95	0	85	15	4	96	78	22

Parameters of the real-time PCR (ct value and melting temperature Tm), and substitutional analysis of the nucleotide positions (NP) 1 to 6 of the pyrograms given as percent (%) of evidence. The various β-lactamases present in the strains are listed in Table 1.

### Discriminative performance of pyrosequencing

Analysis of pyrosequencing results for region 1 and region 2 of all variants revealed that this amplification-sequencing approach was able to assign all tested CTX-M (n=120) variants to the accurate type and group. Region 2 (NP 3 corresponding to Asp240Gly), associated with increased ceftazidime resistance, did not clearly differentiate between all CTX-M-groups on its own. However, a combination of both the group-discriminative nucleotide sequence downstream of +227 (region 1) and the second position related to ceftazidime resistance increased the discriminative performance of the method. 

Naas et al. already described CTX-M characterization by pyrosequencing [33] (in 57 isolates). They sequenced two regions, with one of them being identical to our region 2. However, most of the discriminative NPs identified by Naas et al. are located within the peripheral regions of the sequences, which may impair correct and robust phylogenetic discrimination. Possibly, a combination of all three conserved regions may increase the phylogenetic accuracy.

How far this method is compatible with the commonly used PCR-based multiplex techniques for differentiation of TEM, SHV, or some OXA-variants needs to be evaluated (Figure 2). 

## Conclusions

The developed diagnostic approach focused on the specific molecular determination of CTX-M β-lactamases and a simultaneous discrimination of the sub-groups of this β-lactamase type, to overcome the traditional time consuming phenotypic resistance testing. In order to predict the resistance phenotype more accurately, our focus of interest is mRNA reflecting the expressed genes. We chose this approach since clinical studies have demonstrated that the resistance phenotype, but not the sole detection of a resistance gene, was associated with an impaired clinical outcome, e.g. in *Klebsiella pneumoniae* blood stream infections [35]. Moreover, since usually many transcriptional copies of one gene can be expected in one cell; focusing on mRNA could increase the chance to determine even low cfu/ mL directly from blood samples. However, further work on the usage of EDTA-blood as direct material could be considered to improve the yield of mRNA.

Due to the relative high variability of the CTX-M genes, degenerated primers for RT, and qRT, as well as for pyrosequencing, were used and the suitability and discriminatory performance of two conserved positions within the CTX-M genes were analyzed. The reaction-flow contained a reverse transcription, followed by PCR and pyrosequencing, using one protocol for all isolates and positions respectively. Using this approach no information regarding the expected CTX-M variant is needed, since all sequences are covered by these degenerated primers. 

This work demonstrates the potential of PCR-based verification procedures and how they can be coupled to pyrosequencing to discriminate a wide range of various resistance genes. Our results suggest that the combination of amplification and pyrosequencing can resolve the challenge of continuously emerging new CTX-M variants and the quickly changing local CTX-M epidemiology. In the context of the ongoing fast global spread of emerging novel variants of β-lactamases that are highly exchangeable, even between non-related Gram-negative species, discrimination of pathogens in clinical specimen alone is of minor use and will have to be replaced by direct determination of expressed resistance genes to select the best fitting antibiotic therapy [7,36]. Furthermore, this PCR-based method, coupled to real-time sequencing, can be a fast tool for outbreak analysis, tracing the clonal spread of the resistance genes in hospitals.

## Supporting Information

Figure S1 **Phylogenetic tree of 120 CTX-M variants.** Groups are indicated besides the respective variants.
(TIF)

Figure S2 **Sensorgrams of the qRT.** A) Sensorgram of the qRT run of 45 cycles; the c_t_-values were determined at a threshold of 0.45. B) Melting curve analysis of the PCR-products. Red colored lines indicated the CTX-M-15 that was also used as a calibration standard. Colors are used as indicated on the right site, NTC = negative control reaction without template.
(TIF)

Figure S3 **Melting curve analysis of OXY-β-lactamase.** No PCR-products of OXY-β-lactamase (green and blue lines, technical replicates) could be determined by using the degenerated primer sets for reverse transcription and qPCR. Linearized plasmidal encoded CTX-M-15 gene was used as a positive control (red line). NTC = negative control reaction without template (black line).
(TIF)
