# Supplementary figures and images for: Direct RNA-Based Detection and Differentiation of CTX-M-Type Extended-Spectrum β-Lactamases (ESBL)
Source: PLoS One. 2013 Nov 5;8(11):e80079. doi: 10.1371/journal.pone.0080079 (PMC3818264; doi:10.1371/journal.pone.0080079)

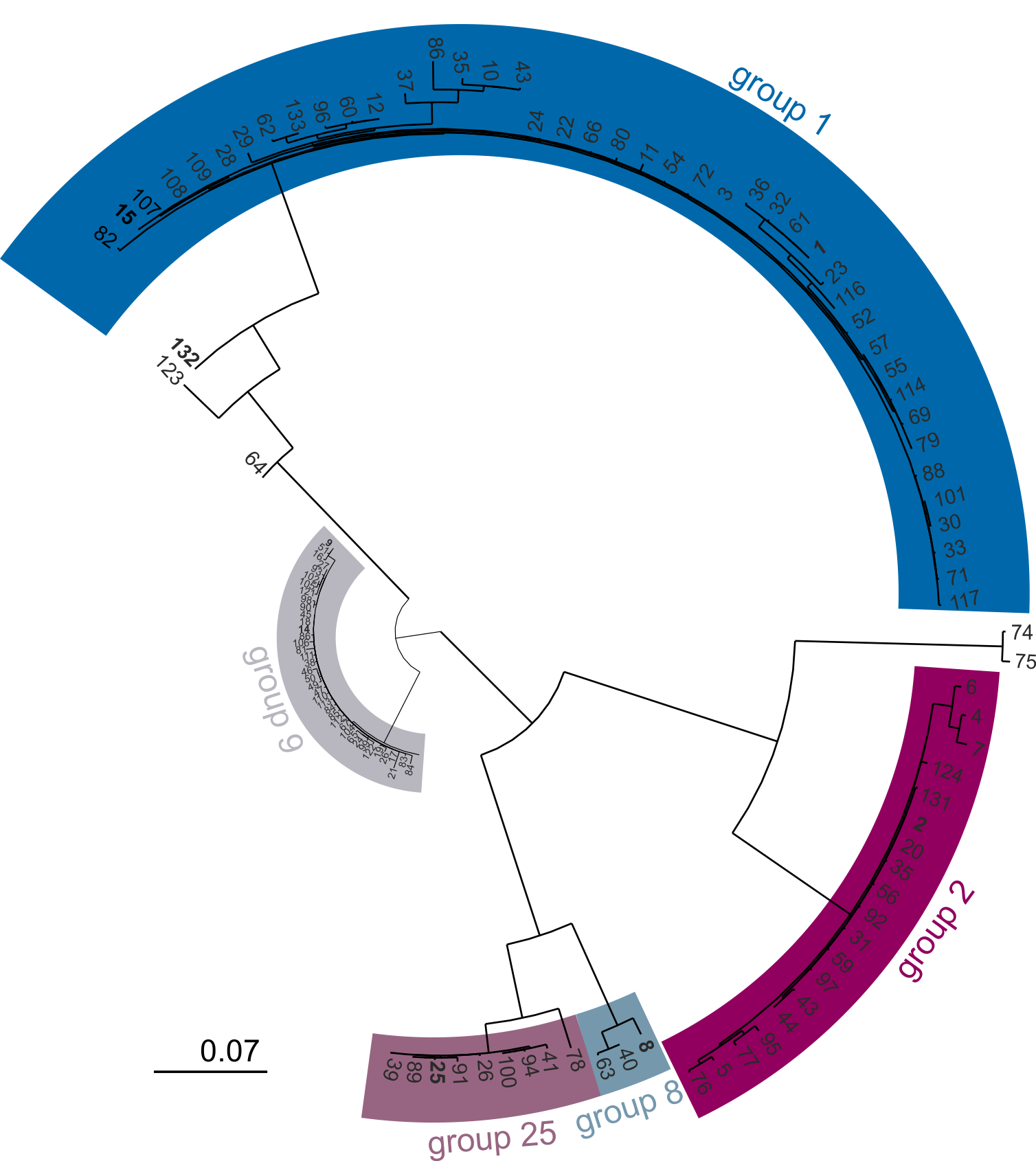

Supplement: Figure S1 — Phylogenetic tree of 120 CTX-M variants. Groups are indicated besides the respective variants. (TIF) [file pone.0080079.s001.tif]

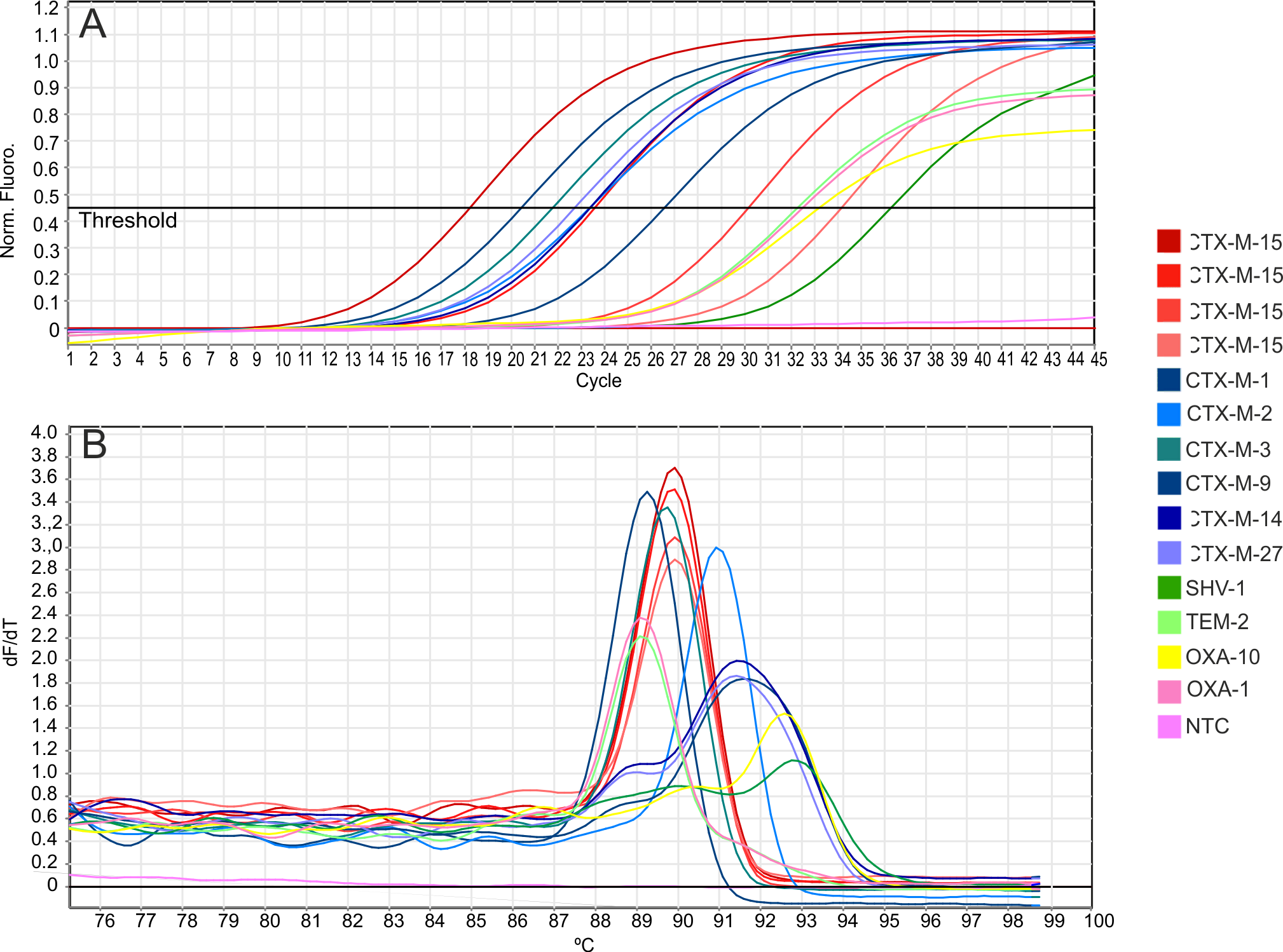

Supplement: Figure S2 — Sensorgrams of the qRT. A) Sensorgram of the qRT run of 45 cycles; the ct-values were determined at a threshold of 0.45. B) Melting curve analysis of the PCR-products. Red colored lines indicated the CTX-M-15 that was also used as a calibration standard. Colors are used as indicated on the right site, NTC = negative control reaction without template. (TIF) [file pone.0080079.s002.tif]

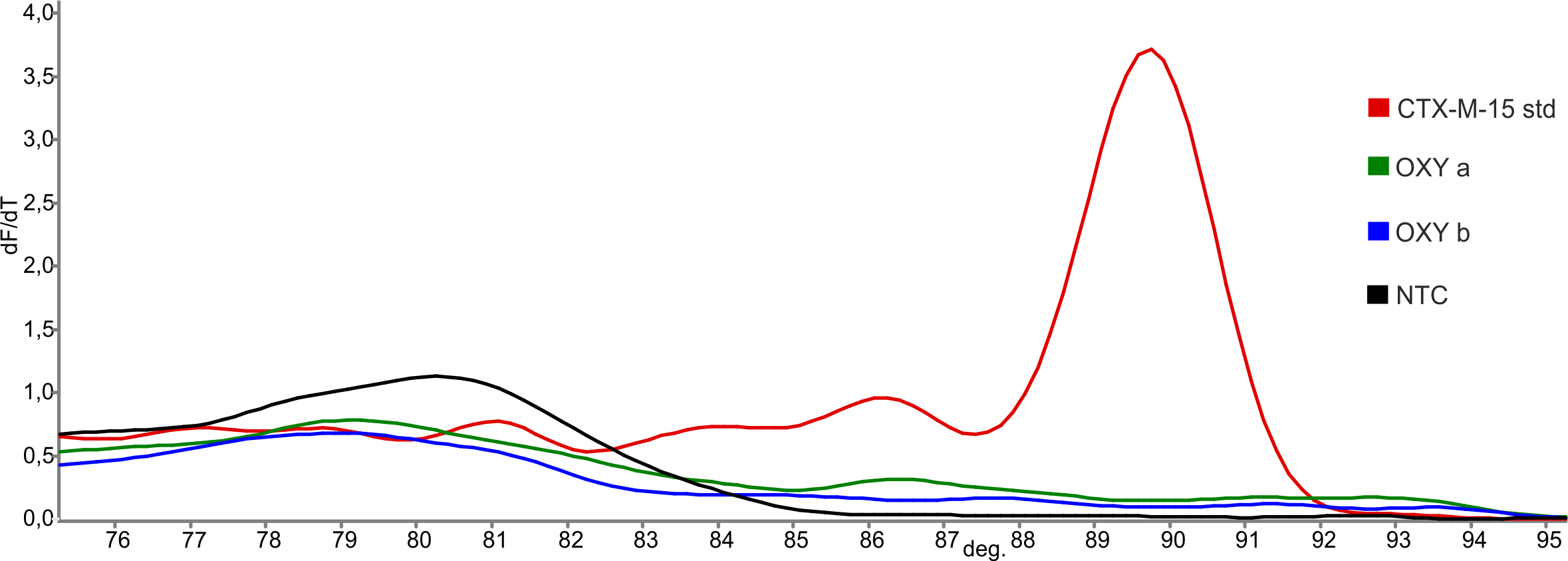

Supplement: Figure S3 — Melting curve analysis of OXY-β-lactamase. No PCR-products of OXY-β-lactamase (green and blue lines, technical replicates) could be determined by using the degenerated primer sets for reverse transcription and qPCR. Linearized plasmidal encoded CTX-M-15 gene was used as a positive control (red line). NTC = negative control reaction without template (black line). (TIF) [file pone.0080079.s003.tif]
